# How leadership behaviors influence the effects of job predictability and perceived employability on employee mental health – a multilevel, prospective study

**DOI:** 10.5271/sjweh.3880

**Published:** 2020-07-01

**Authors:** Lise Fløvik, Cand psychol, Stein Knardahl, Jan Olav Christensen

**Affiliations:** National Institute of Occupational Health, Department of Work Psychology and Physiology, Oslo, Norway

**Keywords:** Key terms direct effect, employee health, job insecurity, moderating, organizational change, psychosocial

## Abstract

**Objectives:**

This study aimed to elucidate the potential moderating effect of fair-, empowering-, and supportive-leadership behaviors on the relationship between job predictability, future employability, and subsequent clinically relevant mental distress.

**Method:**

The study had a full panel, prospective design, utilizing online, self-administered questionnaire data collected at two time points, two years apart. Fair-, empowering-, and supportive-leadership behaviors, job predictability and future employability were measured by the General Nordic Questionnaire for Psychological and Social Factors at Work (QPS_Nordic_). Mental health was measured using the 10-item Hopkins Symptom Checklist (HSCL-10), with cut-off set to >1.85 to identify clinically relevant cases. As data were nested within work units, a multilevel analytic approach was chosen.

**Results:**

Individual-level direct effects: (i) higher levels of job predictability [odds ratio (OR) 0.83, 95% confidence interval (CI) 0.70–0.98], (ii) future employability (OR 0.83, 95% CI 0.74–0.93), (iii) fair- (OR 0.78, 95% CI 0.68–0.91), empowering- (OR 0.77, 95% CI 0.67–0.87), and supportive- (OR 0.71, 95% CI 0.61–0.81) leadership behavior, and (iv) the combination “quality of leadership” (OR 0.69, 95% CI 0.59–0.81) were significantly associated with a lower risk of reporting subsequent mental distress. Work-unit level direct effects: higher work-unit levels of fair- (OR 0.52, 95% CI 0.34–0.80) and empowering- (OR 0.61, 95% CI 0.40–0.94) leadership behaviors and quality of leadership (OR 0.54, 95% CI 0.34–0.87) were significantly associated with a lowered risk of subsequent mental distress. Cross-level interactions: No cross-level interaction effects were shown.

**Conclusions:**

Leadership behaviors did not moderate the effects of job predictability and future employability on mental health. However, employees embedded within work-units characterized by fair, empowering and supportive leadership behaviors had a lower risk of subsequent mental distress.

Contemporary work life is constantly changing, requiring both organizations and employees to adapt (1). Extensive workplace changes such as company restructuring and downsizing have been associated with adverse effects on employee health, work ability and productivity (2, 3). During change, employees’ perceptions of job predictability and future employability may be altered, possibly affecting mental health (4, 5). Mental illness is one of the stronger contributors to work disability worldwide (6). Common psychiatric disorders such as anxiety and depression are amongst the most prevalent (7) and associated with large societal and individual costs (8). Various types of organizational changes (2, 3) (the latter study was partly based on the same cohort as the present study) and psychosocial working conditions have been associated with symptoms of depression and anxiety (9, 10), in particular job security, ie, predictability regarding one’s future job prospects (4, 11). As low job predictability affects an increasingly larger part of the working population (12), there is a pressing need to discern the impact on employee mental health. Moreover, in order to develop effective countermeasures, modifiable factors that may alleviate possible adverse impacts associated with low job predictability must be identified. The present study had two main aims: to (i) determine whether job predictability and future employability predicted employee mental health and (ii) assess the potential moderating effect of fair-, empowering-, and supportive-leadership behaviors at work-unit level on the effect of job predictability and future employability on employee mental health.

Employees experiencing organizational changes, such as restructuring or downsizing, have reported reduced predictability regarding current and future job prospects (4, 5, 13). Reduced predictability has also been shown to persist long after implemented change (14, 15) and is associated with health complaints, turnover, lowered productivity, job satisfaction, and low work engagement both in the short and long term (4, 11). Conversely, increased job predictability has been linked to trust in management, openness towards change, and lowered mental strain (16, 17). As the frequency and extent of organizational changes in contemporary work life are increasing, predictability regarding ongoing work arrangements as well as employability in the future has become a central concern for an increasing number of employees. Much of the change and job-related uncertainty present in contemporary work life may be inevitable, highlighting the significance of identifying factors that can be influenced in order to facilitate a healthy work life (18). However, less is known regarding potential modifiable moderators of the relationship between job predictability and health. If such factors can be pinpointed in the work environment, organizations may influence them through strategic interventions (19).

Leadership is one factor that is amenable to strategic interventions (20, 21). Leadership is defined as the ability and responsibility to guide others in achieving a goal through processes of formal and/or informal influence (22). Various leadership behaviors have been associated with health, coping, productivity, and performance (23, 24). The current study aimed to determine whether fair-, empowering-, or supportive-leadership behaviors moderated prospective effects of job predictability on employee mental health. Fair leadership is characterized by a strong focus on upholding procedural justice and ethics, transparency in decision-making and equal treatment (25). Empowering leaders maintain a strong focus on promoting employee participation, skill development, and enablement (25), while supportive leaders are attentive and considerate towards employees (26). Prior studies have indicated these dimensions to be separate, but related, aspects of leadership (27, 28). Leadership rated low in justice or support has been linked to health complaints, poor social climate, reduced productivity, and sick leave (29). Controversially, higher levels of justice and support have been associated with productivity, organizational commitment and citizenship behavior (30, 31). Acceptance of change and trust in management have also been reported in organizations where employees perceive justice to be prominent (26, 32).

Fair-, empowering-, and supportive-leadership behaviors may represent resources that may help employees cope with challenges associated with work related uncertainty. Support – both instrumental and emotional – and empowerment may help employees take an active role and promote appraisals of change and uncertainty as an opportunity rather than an unmanageable threat. Management operating according to pre-defined agreements may counteract fears of random and unjust treatment in uncertain situations. In addition to the individual perception of one’s superior, the more general perception of leadership within a department or work unit may also influence the individual employee’s sense of predictability and health. Working in environments generally characterized by high levels of these behaviors may prove protective as employees are immersed in a more generally positive work climate independently of their own specific work situation. Despite a widespread interest in the health impact of various aspects of the work environment, most studies of health effects of job predictability have considered only the individual level. However, effects of job predictability on employee mental health may depend on both individual dispositions *and* characteristics of the context, eg, the working conditions within which employees are embedded. As employees within work units share superiors, leadership behavior in particular may constitute such a shared, contextual factor. Thus the present study utilized a multilevel analytical approach in order to take this potential shared group variance into account. In addition, aggregated, group-level predictors (eg, work-unit means) may reduce the influence of individual response characteristics, hence attenuating potential error due to response bias.

## Hypotheses

**Figure 1:**
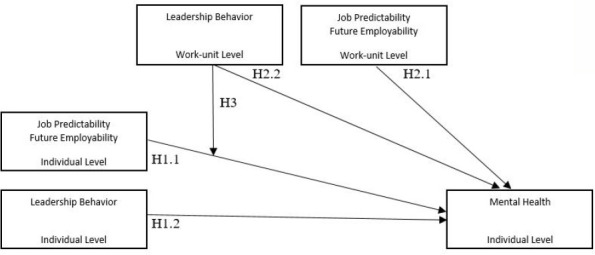
Conceptual, multilevelresearch model displaying the direction of the proposed hypotheses.

Based on the above, three main hypotheses were tested. Figure 1 gives an overview of the hypothesized effects and directions.

*Hypothesis 1: Individual-level direct effects*. We hypothesized job predictability, future employability, and leadership behaviors to predict subsequent mental health.

*Hypothesis 1.1:* Higher levels of (i) job predictability and (ii) future employability at the individual level (ie, level 1) predicts a lower risk of reporting mental distress at follow-up, two years later.

*Hypothesis 1.2:* Higher levels of (i) fair- (ii) empowering-, and (iii) supportive-leadership behavior or (iv) “quality of leadership” (the combination of these three aspects) predicts a lower risk of reporting mental distress at follow-up.

*Hypothesis 2: Work-unit level direct effect*. The second hypothesis assessed the work-unit level, direct effect of job predictability, future employability and leadership behaviors on subsequent mental health - ie, the effects of work-unit levels of predictability, employability, and leadership behaviors on individual mental health.

*Hypothesis 2.1:* Employees embedded within work units characterized by higher levels of job predictability and future employability exhibit a lower risk of reporting subsequent mental distress.

*Hypothesis 2.2:* Work-unit employees characterized by higher levels of (i) fair-, (ii) empowering-, (iii) or supportive-leadership behaviors or the combination of these, (iv) “quality of leadership”, exhibit a lower risk of reporting subsequent mental distress.

*Hypothesis 3: Cross-level interaction effect*. The third hypothesis assessed the potential cross-level interaction of leadership behaviors at work-unit level with job predictability and future employability in predicting subsequent mental health.

*Hypothesis 3.1:* Work-unit levels of fair- (i), empowering- (ii), supportive-leadership behaviors (iii) or the combination of these, “quality of leadership” (iv), moderate the prospective relationship between the individual employee’s job predictability or future employability and subsequent mental distress.

## Method

### Procedure and participants

The study was a part of the project “The New Workplace: Work, Health and Participation in Working Life”, administered by the Norwegian National Institute of Occupational Health (STAMI). All data were collected by self-administered online questionnaires, covering a wide range of demographic-, work- and health-related variables. The study had a full-panel, repeated-measures design. Baseline data were collected during 2009–2013, with follow-up after two years. A previous study investigated the effects of leadership and predictability on mental distress with data from the current project, with baseline data collected during 2004–2009 (10). To compare results, avoid overlapping samples, and possibly yield a sample more representative of contemporary working life, we selected subjects recruited for the baseline sample after 2009.

The participating organizations contacted STAMI with a general request for aid in a work environment survey or in response to an invitation to participate in the project published on STAMI’s home page. All participating organizations were located in Norway and included a wide variety of professions in both the public and private sector. All current employees and managers in the participating companies were invited to take part in the study. In total, 8140 employees were invited at baseline. Of these, 5166 (63.6%) responded at baseline, while 3405 (65.7%) also participated at follow-up. Inclusion criteria was completing the outcome measure at baseline, while dropout was defined as not having completed the outcome measure at follow-up. For further details, see table 1.

**Table 1:** Sample characteristics. Characteristics of baseline sample and prospective sample. Inclusion criteria was the completion of Hopkins Symptom checklist (HSCL-10).

	Invited subjects		Baseline sample		Prospective sample	
		
	N	%	N	%	N	%
Sex						
Female			2442	47.3	1688	49.6
Male			2724	52.7	1717	50.4
Total	8140	100	5166		3405	
Missing			2944	36.4		
Age (years)						
<29–39			1910	36.9	1164	34.2
39–49			1667	32.3	1141	33.5
>49			1589	30.7	1100	32.3
Total			5166		3405	
Skill level (years)						
>15			1610	31.1	1130	33.2
13–15			664	12.8	249	10.2
>10–12			2371	45.8	1523	44.7
Unspecified			533	10.3	403	11.8
Workplace						
Public sector			4251	82.3	2749	80.7
Private sector			915	17.7	656	19.3

### Variables

*Predictor: Job predictability and future employability*. Job predictability and future employability were assessed by the General Nordic Questionnaire for Psychological and Social Factors at Work (QPS_Nordic_) (25). Job predictability was measured by three items assessing to what degree employees know what tasks, co-workers, and superiors they can expect for the next month in their current job. Future employability was measured by the work factor “predictability of the next two years”, with two items assessing the employee’s confidence that they possess the competence and abilities needed to acquire an attractive job in two years. Responses on all items in both predictors were given on a 5-point Likert scale (range 1=very seldom to never to 5=very often or always). For both scales, a mean score was calculated. At baseline, Cronbach’s α was 0.64 for job predictability and 0.73 for future employability. See the supplementary material for all included items (www.sjweh.fi/show_abstract.php?abstract_id=3880).

*Predictor and moderator: Leadership behaviors*. The three dimensions of leadership behaviors (fairness, empowerment and support) were assessed utilizing the QPS_Nordic_ (25). The mean of the three leadership factors was also calculated to reflect a more general scale of leadership, labelled “quality of leadership”, as specified in the QPS_Nordic_ manual (25). Responses on all items were given on a 5-point Likert scale, ranging from 1=very seldom or never to 5=very often or always. At baseline, Cronbach’s α was as follows fair leadership=0.86, empowering leadership=0.88, support from superior=0.85, and quality of leadership=0.90.

*Outcome: Clinically relevant mental distress*. The 10-item Hopkins Symptom Checklist (HSCL-10) (33) was employed to assess clinically relevant mental distress. HSCL-10 is a self-report instrument for assessing symptoms of mental distress (ie, symptoms of anxiety and depression) utilized in both clinical- and population studies (33, 34). For each item, a statement is presented and respondents are to report how the statement match their own experience within the last seven days. Responses are measured on a 4-point Likert scale ranging from 1=not at all to 4=very much. Cronbach’s α at baseline was 0.86. Based on the ten items, a mean score was calculated. To identify clinically relevant cases, a cut-off was set to ≥1.85 and the reliability and validity of HSCL-10 have been demonstrated repeatedly (33, 34).

### Potential confounders

Age, sex, skill level, and organizational change were included as potential confounders in all analyses. Age was arranged into four groups: (i) <29–39, (ii) >39–49, (iii) >49–59 and (iv) >59 years. Skill level categories were created using the International Standard Classification of Occupations (ISCO-88). The different categories reflect the number of years of formal education typically required to qualify for a certain profession: (i) ≤12, (ii) 13–15, and (iii) >15 years. Analyses were also adjusted for a number of distinct types of organizational changes as change may influence health by mechanisms not related to predictability.

### Statistical analyses

*Multilevel modelling*. Multilevel logistic regressions (generalized linear mixed effects regression, GLMER) were employed to estimate the prospective associations due to the dichotomous outcome and the dataset nested structure. The organizations differed substantially in size and scope, ranging from one-unit organizations to organizations with several work units spread out over large geographical locations. As a result, we hypothesized employees within work units would share more contextual variables than employees within the total organization, and work-unit membership was designated as the grouping variable.

The multilevel approach takes into consideration the clustering of measurements. Not accounting for shared variance amongst measurements, eg, due to a shared environment, violates the assumption of independence, which may bias estimates, eg, underestimate standard errors (35) and increase the risk of type I error in the presence of a high within-group correlation of measurements, ie, high intra-class correlation (ICC) (36). As ICC indicate the degree to which measurements correlate within groups and variance is explained by between-group characteristics, ICC were estimated to assess the appropriateness of applying a multilevel approach. In the present sample, ICC were as follows: fair leadership=0.101, empowering leadership=0.071, and support from superior=0.082. An ICC of 0.05 indicates a small- to medium-sized group effect and has been suggested as a threshold for applying multilevel analyses (37). However, prior studies have selected ICC of 0.01 to indicate statistically significant group-level effects in data (38). As the present ICC were in the range of 0.10–0.07, we considered it appropriate to apply a multilevel assessment.

Multilevel modelling provides the possibility to explore both individual- and work-unit level direct effects and cross-level interaction effects (35). To assess the associations at the individual- and work-unit level separately, ie, to separate the effects, the individual level predictor was group mean centered, while the predictor at work-unit level was the aggregated work-unit mean (39). When group-mean centering scores at the individual level, the mean score of each employees’ respective work unit is subtracted from each employee’s individual score. The shared work-unit variance is then removed from the individual score, making the individual score uncorrelated with the work-unit mean. Thus, group-mean centering disentangles the effect of the predictor at the individual- and work-unit level and the effects can then be considered separately (39). In the multilevel model, variance parameters are estimated for both intercept and regression coefficient (slope) for all sample groups, in this case work units. For best model fit, both fixed and random effects may be incorporated to model variability. Fixed effects refer to effects equal for all groups, while random effects refer to effects that vary between groups. In a random intercept only model, the group intercept (intercept for each work-unit) is allowed to vary, while the regression coefficient (slope) is held constant for all groups. In a random intercept and slope model, both intercept and slope vary across groups (35). In the present analyses, both random intercept only and random intercept and random slope models were tested. For each model, likelihood ratio tests were used to ascertain best model fit. The random intercept only provided the best model fit for all models, except when including interaction terms, as the purpose was to estimate the effect of work unit levels of leadership behavior on variability of the slope at the individual level.

All analyses pertaining to direct and interaction effects were run in two steps: Model I was adjusted for the potential confounders age, sex, skill level and organizational changes, and model II was additionally adjusted for mental distress at baseline. Post-hoc analyses of the potential moderating effect of leadership behaviors at the individual-level were all adjusted for baseline mental distress. All analyses were run using IBM SPSS Statistics, version 25.0 (IBM Corp, Armonk, NY, USA) and R, version 3.4.4 (R Foundation for Statistical Computing, Vienna, Austria). The level of statistical significance was set to P<0.05.

## Results

### Non-response and attrition analysis

Non-response was negatively associated with professions with unspecified/no formal requirements [odds ratio (OR) 0.61, 95% confidence interval (CI) 0.37–0.99]. Attrition analysis showed women were more likely to participate at follow-up (OR 1.31, 95% CI 1.18–1.46). Working in professions with >10 years of formal requirements (OR 4.33, 95% CI 1.95–9.63), 10–12 years (OR 1.20, 95% CI 1.05–1.37) and 13–15 years (OR 1.21, 95% CI 1.05–1.40) were also associated with participating at follow-up (table 2).

**Table 2:** Non-response and attrition analyses. Non-response defined as not completing HSCL-10 at baseline. Attrition defined as completing HSCL-10 at baseline, but not at follow-up. Statistically significant odds ratios (OR) and corresponding confidence intervals (CI) are written in **bold font**.

	Non-response		Attrition	
	
	OR	95% CI	OR	95% CI
Sex				
Male	1	1	1	1
Female	1.10	0.88–1.38	**1.31**	**1.18–1.46**
Age (years)				
<29–39	1	1	1	1
39–49	0.84	0.64–1.10	1.12	0.97–1.29
>49	0.90	0.62–1.30	0.96	0.80–1.15
Skill level (years)				
>15	1	1	1	1
13–15	1.28	0.91–1.80	**1.21**	**1.05–1.40**
<10–12	1.06	0.81–1.39	**1.20**	**1.05–1.37**
Unspecified	0.61	0.37–0.99	1.19	0.99–1.43
Workplace				
Private sector	1	1	1	1
Public sector	0.79	0.60–1.04	**0.45**	**0.41–0.53**
Mental distress			0.99	0.97–1.01
Job predictability			**1.18**	**1.01–1.26**
Future employability			0.99	0.94–1.04

### Hypothesis 1: individual-level direct effects

*Predictability*. Baseline adjusted analyses showed higher levels of job predictability and future employability at the individual level to be statistically significantly associated with lower risk of clinically relevant distress two years later, OR 0.84, 95% CI 0.71–0.99 and 0.82, 95% CI 0.73-0.93, respectively (table 3). For non-baseline adjusted analyses, see supplementary material, table S1.

**Table 3:** Individual level direct effects – baseline adjusted. Prospective direct effects of job predictability, future employability and leadership behaviors at the individual level on subsequent clinically relevant mental distress two years after. Displaying the results of hypotheses 1.1 and 1.2. Adjusted for mental distress at baseline, age, sex, skill level, and organizational changes. Statistically significant odds ratios (OR) and corresponding confidence intervals (CI) are written in **bold font**.

	Individual level	

	OR	95% CI
Predictability		
Job predictability one month	**0.84**	**0.71–0.99**
Employment predictability two years	**0.82**	**0.73–0.93**
Leadership		
Quality of leadership total	**0.70**	**0.60–0.83**
Support from superior	**0.71**	**0.62–0.82**
Empowering leadership	**0.77**	**0.67–0.88**
Fair leadership	**0.81**	**0.69–0.94**

*1.2 Leadership*. Baseline-adjusted analyses showed all included leadership behaviors at the individual level to be statistically significantly associated with a lower risk of reporting clinically relevant mental distress at follow-up: fair- (OR 0.81, 95% CI 0.69–0.94), empowering- (OR 0.77, 95% CI 0.67–0.88), and supportive- (OR 0.71, 95% CI 0.62–0.82) leadership behavior and quality of leadership (OR 0.70, 95% CI 0.60–0.83). See table 3. For non-baseline adjusted analyses, see supplementary table S1.

### Hypothesis 2: work-unit level direct effect

*Predictability*. Baseline adjusted analyses showed no statistically significantly prospective associations between work-unit levels of job predictability or future employability and mental distress. See table 4 for further details. For non-baseline adjusted analyses, see supplementary table S2.

**Table 4:** Work-unit level direct effects – baseline adjusted. Prospective direct effects of job predictability, future employability and leadership behaviors at work-unit level on subsequent clinically relevant mental distress two years after. Displaying the results of hypotheses 2.1 and 2.2. Adjusted for mental distress at baseline, age, sex, skill level, and organizational changes. Statistically significant odds ratios (OR) and corresponding confidence intervals are written in **bold font**.

	Work-unit Level	

	OR	95% CI
Predictability		
Job predictability one month	0.68	0.42–1.12
Employment predictability two years	0.89	0.68–1.24
Leadership		
Quality of leadership total	**0.59**	**0.36–0.96**
Support from superior	0.86	0.55–1.35
Empowering leadership	**0.64**	**0.41–1.00**
Fair leadership	**0.56**	**0.36–0.87**

*Leadership*. Baseline adjusted analyses showed higher levels of fair- and empowering leadership behaviors and quality of leadership at work-unit level to be statistically significant associated with lower risk of reporting clinically relevant mental distress at follow-up. OR were as follows: fair- (OR 0.56, 95% CI 0.36–0.87), empowering- (OR 0.64, 95% CI 0.41–1.00) leadereship behavior, and quality of leadership (OR 0.59, 95% CI 0.36–0.96). Work-unit levels of supportive leadership behaviors showed no statistically significant association with subsequent mental distress (table 4). For non-baseline adjusted analyses, see supplementary table S2.

### Hypothesis 3: Cross-level interaction effects

Baseline adjusted analyses showed no statistically significant cross-level interaction effect of leadership behaviors at work-unit level (table 5). For non-baseline adjusted analyses, see supplementary table S3.

**Table 5:** Cross-level interaction effects – Baseline adjusted. The impact of work-unit level of leadership behaviors on the prospective effect of job predictability and employability on subsequent clinically relevant mental distress. Displaying the results of hypothesis 3. Analyses adjusted for mental distress at baseline, age, sex, skill level and organizational change. Main effects from moderated regressions not shown.

	Cross-level interaction	

	OR	95% CI
Quality of leadership		
Job predictability one month × quality of leadership	0.82	0.44–1.53
Employment predictability two years × quality of leadership	0.89	0.57–1.38
Support from superior		
Job predictability one month × support from superior	0.68	0.37–1.23
Employment predictability two years × support from superior	0.91	0.61–1.35
Empowering leadership		
Job predictability one month × empowering leadership	0.95	0.55–1.64
Employment predictability two years × empowering leadership	0.95	0.64–1.40
Fair leadership		
Job predictability one month × fair leadership	0.98	0.53–1.82
Employment predictability two years × fair leadership	0.86	0.58–1.29

## Discussion

The present results demonstrated higher predictability regarding one’s present or future job prospects at the individual level to be associated with a lower risk of subsequent mental distress. These results are in line with prior studies linking higher levels of job predictability and future employability with positive effects on health (16) and low predictability to health complaints (11). Work-unit levels of job predictability and future employability were not associated with subsequent mental health, which indicate that generally high levels of unpredictability within work-units does not necessarily influence the mental health of individual employees. Thus, the health effects associated with work-related uncertainty seem less influenced by the general situation in one’s respective work-unit, but rather depend on each employee’s appraisal of their own situation.

The present results also showed higher levels of fair-, empowering- and supportive-leadership behaviors – and the combination of these, ie, “quality of leadership” – at the individual level were associated with a lower risk of reporting mental distress at follow-up. Work units characterized by higher levels of fair and empowering leadership behaviors and the combination of these were also found to have a protective, prospective effect on employee mental health. However, the effect of supportive leadership behaviors were no longer significant when measured at work-unit level. These results are in line with and adds to the findings of prior studies linking leadership characterized by fairness, empowerment and support to employee health (23, 24). Hence, results show that both individual and work-unit perception of leadership influence mental health prospectively. One notable exception was supportive leadership, which did not exhibit statistically significant associations at the work-unit level. Compared to fairness and empowerment, one may speculate that support from one’s immediate superior constitutes more of a personal- and individual-level construct. Fair- and empowering-leadership behaviors consists of a range of aspects pertaining to the individual employee, but may also represent more general conducts defining organizational values and leadership expectations, which influence and applies to all member of a specific group or work-unit. Whereas support from one’s superior may be mainly individual focused, representing a unique process and content for each individual employee depending on their needs and their superior’s ability to meet these.

No significant cross-level interaction effect of leadership behaviors on the effect of job predictability and future employability on mental health was shown. These results contrast prior reporting leadership high in support and fairness to moderate the effect of workplace stressors on outcomes such as employee health, cooperation, and performance (40, 41). However, not all prior studies have shown such leadership dimensions to buffer the effect of stressors (42, 43). In sum, the included leadership behaviors did not moderate the effect of predictability on mental health; however, all leadership behaviors had a direct prospective, protective effect on subsequent mental health, at both the individual and work-unit level. Hence, management may focus efforts on promoting fair, empowering, and supportive superiors in order to promote employee health. Furthermore, one may speculate that the positive impact of leadership may partially stem from such leadership promoting a predictable and secure work environment. Hence, rather than leadership behaviors moderating the effects of low predictability, predictability may be one of the factors mediating the effects of leadership on mental health. A thorough investigation was outside the scope of the present study, however, post-hoc random intercept linear regressions were run to examine the effect of leadership behaviors on subsequent job predictability and employability. The results showed a statistically significant prospective effect of all leadership behaviors on both job predictability and future employability, providing preliminary support to this notion. For job predictability unstandardized betas were within the range of 0.02–0.04, for future employability betas ranged from 0.05–0.09. For further details, see supplementary table S4.

It should be noted that cross-level interaction effects may be difficult to detect and could be affected by limitations of the present study, such as timeframe, level of measurement, lower statistical power due to fewer observations at work-unit level and variables not assessed in the analyses (39). Prior studies have found the level of analysis to influence the detection of the moderating effect of leadership on health, while others have found the moderating effect of supportive leadership only to be present in certain subgroups of the sample (44). On this note, in addition to being a group-level characteristic, leadership may also represent a unique relationship between supervisor and employee. The present results show leadership behaviors at the individual level have a direct effect on employee health, hence, leadership measured at the individual level could potentially capture different aspects of leadership, which group-level measures may not detect. In order to assess the potential moderating effect of leadership behaviors at the individual level, post-hoc individual-level interaction analyses were run. These did not show a significant moderation effect for any of the included leadership behaviors and were thus in line with the cross-level interaction analyses. For further details of the results of the individual level interaction analyses, see supplementary table S5. Although no difference in individual- or cross-lever interaction effects were shown in the present results, leadership represent both a shared and individual process, hence measuring the effect at both the individual- and group-level may more accurately comprise the total effect of leadership, as shown in the significant direct effects at both the individual- and work-unit level.

### Methodological considerations

The timespan between measurements in the study may have influenced results, with transient health effects emerging and resolving within the two years before follow-up not detected. Effect estimates in the present study may be underestimated due to inherent limitations of estimating complex processes at discrete time points. Furthermore, clinically relevant mental distress was identified by a cut-off criterion (34), leaving health effects at the subclinical levels undetected and effects underestimated. Although clinical cut-off is a strict criterion, we utilized this in order to identify work exposures, which may have profound impact on employee functioning and quality of life both at and outside of work. This is especially pertinent given the current challenge of mental illness being one of the leading causes of disability, sick leave, and production losses (12). Not adjusting for type of job contract may also have influenced results as one might speculate type of contract to be associated with differences in job predictability (45). Sample composition and self-selection may also have influenced results as organizations were invited to sign up for participation themselves.

Attrition analyses linked lower job predictability to dropout, which may compromise the generalizability of the results. Employees working in professions requiring <15 years of formal education were also less likely to participate at follow-up. These are also the professions reporting the lowest job predictability and future employability and which seem the most affected by technological innovations such as automatization (12). We conducted a post-hoc random intercept linear regression analysis to examine the associations between skill level and job predictability and future employability. These results showed workers employed in professions requiring <15 years of formal education reported significantly lower levels of job predictability and future employability (supplementary table S6). Thus, at follow-up, there was a significant drop in responses from the group of workers potentially most affected by the structural changes in modern day work life.

Employees suffering from mental distress at baseline may experience their own future as more uncertain, hence reverse causality may be present. A set of post-hoc random intercept linear regressions were run to assess the prospective associations of clinically relevant mental distress and job predictability and future employability. The results showed mental distress to be associated with lower levels of job predictability and future employability at follow-up (supplementary table S7), hence reverse causality may be present at the individual level. However, prospective analyses adjusting for baseline levels of the outcome should be less sensitive to reverse causality bias as the association of the outcome with the exposure at baseline is partialled out. At work-unit level, reverse causality is less likely even when not baseline adjusted due to the aggregated predictor. Furthermore, perceived predictability and employability possibly influence employees’ ratings of superiors. Present post-hoc analyses showed baseline levels of predictability to predict perceived leadership behaviors (supplementary table S8). Future studies assessing the effects of job predictability and leadership may explore this further.

As data were collected by questionnaires, common method and reporting biases may have influenced results (46). However, the multilevel approach should diminish the influence of such bias as responses are aggregated at the work-unit level, which minimizes the effect of individual response bias (35, 39). Moreover, it is equally important to be aware that non-significant effects at the work-unit level do not prove the existence of reporting bias at the individual level, as some relationships are primarily individual-level phenomena. Non-significant effects at work-unit level may also be due to significantly fewer observations at this level, which attenuates the statistical power (47). The question also remains whether the unit of aggregation is appropriate for the phenomena in question, as different characteristics of work may be shared at different levels, such as, eg, work unit, organization, or job type.

### Future perspectives

The present results show uncertainty regarding one’s present and future job prospects may affect mental health to the extent of clinical relevance. In order to alleviate the straining effects of low predictability, identification of protective factors is essential. Although the present results did not show a moderating effect of leadership behaviors on the effect of job predictability on mental health, a direct effect at both individual- and work-level was shown. The present results suggest that leadership behaviors might be a relevant factor in preventing mental distress, possibly by reducing both short- and long-term uncertainty in the workplace.

## Supplementary material

Supplementary material
