# Supplementary material for: How leadership behaviors influence the effects of job predictability and perceived employability on employee mental health – a multilevel, prospective study
Source: Scand J Work Environ Health. 2020 Jul 1;46(4):392–401. doi: 10.5271/sjweh.3880 (PMC8506305; doi:10.5271/sjweh.3880)
Supplement: Supplementary material [file SJWEH-46-392-S001.pdf]

# How leadership behaviors influence the effects of job predictability and perceived employability on employee mental health – a multilevel, prospective study [1]

by Lise Fløvik, Cand psychol,[2] Stein Knardahl, PhD, Jan Olav Christensen, PhD

1. *Supplementary material*
2. *Correspondence to: Lise Fløvik, Department of Work Psychology and Physiology, National Institute of Occupational Health, P.O. Box 8149 Dep, N-0033 Oslo, Norway. [E-mail: lise.flovik@stami.no]*

**Table S1. Individual level direct effects of job predictability, future employability and leadership behaviors on subsequent mental health – Non-baseline adjusted**

Individual-level direct effects of job predictability, future employability and leadership behaviors on clinically relevant mental distress two years after. Displaying the results of hypothesis 1.1 and 1.2.
Adjusted for age, sex, skill level and organizational change. Not adjusted for mental distress at baseline.

| | **Individual Level** | |
|---|---|---|
| | **OR** | **95% CI** |
| **Predictability** | | |
| Job Predictability One Month | **0.74** | **0.64-0.85** |
| Employment Predictability Two Years | **0.76** | **0.69-0.85** |
| **Leadership** | | |
| Quality of Leadership Total | **0.53** | **0.46-0.61** |
| Support from Superior | **0.57** | **0.50-0.64** |
| Empowering Leadership | **0.64** | **0.57-0.72** |
| Fair Leadership | **0.61** | **0.53-0.69** |

Note: OR: Odds ratio. Statistically significant odds ratios and corresponding confidence intervals are written in **bold font.**

**Table S2. Work-unit level direct effects of job predictability, future employability and leadership behaviors on subsequent mental health – Non-baseline adjusted**

Work-unit level prospective direct effects of job predictability, future employability and leadership behaviors on clinically relevant mental distress two years after. Displaying the results of hypothesis 2.1 and 2.2.
Adjusted for age, sex, skill level and organizational change. Not adjusted for mental distress at baseline.

| | Work-unit Level | |
|---|---|---|
| | OR | 95% CI |
| **Predictability** | | |
| Job Predictability One Month | **0.59** | **0.39-0.91** |
| Employment Predictability Two Years | 0.73 | 0.49-1.07 |
| **Leadership** | | |
| Quality of Leadership Total | **0.51** | **0.33-0.79** |
| Support from Superior | 0.72 | 0.48-1.06 |
| Empowering Leadership | **0.54** | **0.37-0.78** |
| Fair Leadership | **0.55** | **0.37-0.80** |

Note: OR: Odds ratio. Statistically significant odds ratios and corresponding confidence intervals are written in **bold font.**

**Table S3. Cross-level interaction effect of leadership behaviors on the effect of job predictability and future employability on subsequent mental health– Non-baseline adjusted**

The impact of work-unit level of leadership behaviors on the prospective effects of job predictability and employability on clinically relevant mental distress two years later. Analyses adjusted for age, sex, skill level and organizational change. Not adjusted for mental distress at baseline.

Main effects from moderated regressions not shown.

| | **Cross-level Interaction** | |
|---|---|---|
| | **OR** | **95% CI** |
| **Quality of Leadership** | | |
| Job Predictability One Month x Quality of Leadership | 1.11 | 0.64-1.93 |
| Employment Predictability Two Years x Quality of Leadership | 1.05 | 0.72-1.54 |
| **Support from Superior** | | |
| Job Predictability One Month x Support from Superior | 0.76 | 0.44-1.31 |
| Employment Predictability Two Years x Support from Superior | 1.10 | 0.77-1.55 |
| **Empowering Leadership** | | |
| Job Predictability One Month x Empowering Leadership | 0.89 | 0.52-1.51 |
| Employment Predictability Two Years x Empowering Leadership | 1.01 | 0.72-1.41 |
| **Fair Leadership** | | |
| Job Predictability One Month x Fair Leadership | 1.11 | 0.64-1.93 |
| Employment Predictability Two Years x Fair Leadership | 1.03 | 0.73-1.46 |

Note: OR: Odds ratio. Statistically significant odds ratios and corresponding confidence intervals are written in **bold font.**

**Table S4. Prospective effect of leadership behaviors on job predictability and future employability**

Post-hoc analyses of the prospective, individual-level effect of leadership behaviors on job predictability and perceived future employability two years later. Adjusted for mental distress at baseline, age, sex, and skill level.

| | b | 95% CI |
|---|---|---|
| **Fair Leadership** | | |
| Job Predictability One Month | **0.04** | **0.01-0.07** |
| Employment Predictability Two Years | **0.05** | **0.00-0.09** |
| **Empowering Leadership** | | |
| Job Predictability One Month | **0.02** | **0.00-0.05** |
| Employment Predictability Two Years | **0.09** | **0.05-0.13** |
| **Support from Superior** | | |
| Job Predictability One Month | **0.04** | **0.01-0.06** |
| Employment Predictability Two Years | **0.08** | **0.04-0-12** |

Note: b: Unstandardized beta. Statistically significant beta and corresponding confidence intervals are written in **bold font.**

**Table S5. Individual-level interaction effects – Baseline adjusted**

The impact of leadership behaviors at the individual level on the prospective effect of job predictability and employability on subsequent clinically relevant mental distress. Analyses adjusted for mental distress at baseline, age, sex, skill level and organizational change.

Main effects from moderated regressions not shown.

| | Individual-level Interaction | |
|---|---|---|
| | **OR** | **95% CI** |
| **Quality of Leadership** | | |
| Job Predictability One Month x Quality of Leadership | 0.98 | 0.80-1.19 |
| Employment Predictability Two Years x Quality of Leadership | 0.99 | 0.86-1.14 |
| **Support from Superior** | | |
| Job Predictability One Month x Support from Superior | 1.02 | 0.85-1.21 |
| Employment Predictability Two Years x Support from Superior | 1.00 | 0.89-1.13 |
| **Empowering Leadership** | | |
| Job Predictability One Month x Empowering Leadership | 0.96 | 0.81-1.15 |
| Employment Predictability Two Years x Empowering Leadership | 1.01 | 0.90-1.14 |
| **Fair Leadership** | | |
| Job Predictability One Month x Fair Leadership | 1.00 | 0.83-1.19 |
| Employment Predictability Two Years x Fair Leadership | 0.97 | 0.85-1.10 |

Note: OR: Odds ratio. Statistically significant odds ratios and corresponding confidence intervals are written in **bold font.**
Organizational changes: Restructuring, downsizing, layoffs, partial closure, partial outsourcing and M&A's.

**Table S6. Prospective effect of skill level on job predictability and future employability**
Post-hoc analyses of the prospective, individual-level direct effect of skill level requirements on job predictability and future perceived employability two years later. Adjusted for mental distress at baseline, age, and sex.

| | b | 95% CI |
|---|---|---|
| **Skill level >15 years** | | |
| Job Predictability One Month | - | - |
| Employment Predictability Two Years | - | - |
| **Skill level 13-15 years** | | |
| Job Predictability One Month | **-.09** | **-0.18, -0.01** |
| Employment Predictability Two Years | **-.15** | **-0.27, -0.03** |
| **Skill level <10-12 years** | | |
| Job Predictability One Month | **-.36** | **-0.44, -0.29** |
| Employment Predictability Two Years | **-.23** | **-0.33, -0.14** |
| **Skill level Unspecified** | | |
| Job Predictability One Month | **0.17** | **0.09-.26** |
| Employment Predictability Two Years | **0.34** | **0.21-.46** |

Note: b: Unstandardized beta. Statistically significant beta and corresponding confidence intervals are written in **bold font.**

**Table S7. Prospective effects of mental distress on job predictability and future employability**

Post-hoc analyses of the individual-level prospective direct effect of clinically relevant mental distress on job predictability and perceived future employability two years later. Adjusted for age, sex, and skill level.

| | b | 95% CI |
|---|---|---|
| **Clinically Relevant Mental Distress** | | |
| Job Predictability One Month | **-0.07** | **-0.15, -0.01** |
| Employment Predictability Two Years | **-0.15** | **-0.26, -0.04** |

Note: b: Unstandardized beta. Statistically significant beta and corresponding confidence intervals are written in **bold font.**

**Table S8. Prospective effects of job predictability and future employability on leadership behaviors**

Post-hoc analyses of the prospective individual-level direct effect of job predictability and perceived future employability on leadership behaviors two years later. Adjusted for mental distress at baseline, age, sex, and skill level.

| | b | 95% CI |
|---|---|---|
| **Job Predictability One Month** | | |
| Fair Leadership | **0.07** | **0.03-0.11** |
| Empowering Leadership | **0.06** | **0.02-0.10** |
| Supportive Leadership | **0.08** | **0.04-0.11** |
| **Perceived Future Employability** | | |
| Fair Leadership | **0.03** | **0.00-0.05** |
| Empowering Leadership | **0.03** | **0.01-0.06** |
| Supportive Leadership | 0.02 | -0.00-0.05 |

Note: b: Unstandardized beta. Statistically significant beta and corresponding confidence intervals are written in **bold font**

**Figure S1.** Directed Acyclic Graph (DAG) visualizing the hypothesized effects of the confounding age, sex, skill level and organizational change on mental health.

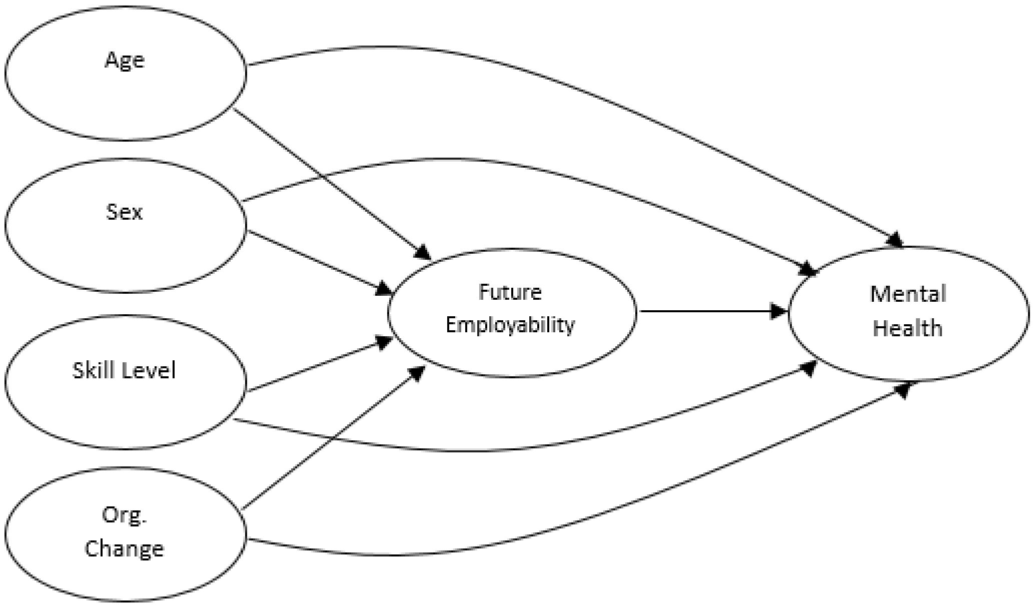


**Figure S2.** Directed Acyclic Graph (DAG) visualizing the hypothesized effects of the confounding age, sex, skill level and organizational change on mental health.

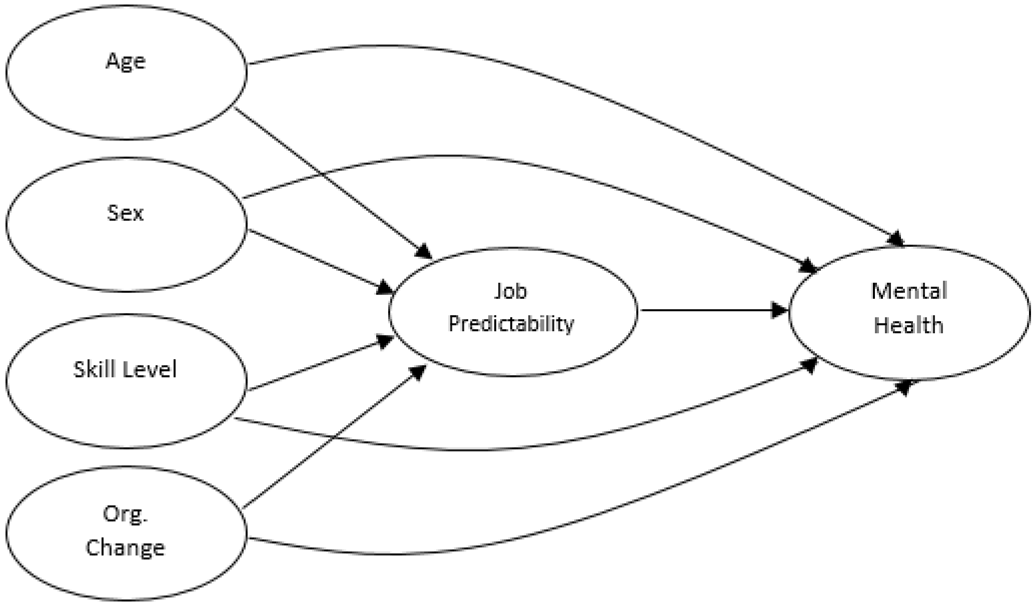

## $QPS_{Nordic}$ Items:

Job Predictability:

1. Do you know in advance what kind of tasks to expect a month from now?
2. Do you know in advance who will be you co-workers a month from now?
3. Do you know in advance who will be your superior a month from now?

Future Employability

1. Do you know what is required in order for you to get a job that you consider attractive in two years?
2. Do you know what has to be learned an which new skills have to be acquired in order for you to maintain a job that you consider attractive in 2 years?

Fair Leadership:

1. Dies you immediate superior distribute the work fairly and impartially?
2. Does your immediate superior treat the workers fairly and equally?
3. Is the relationship between you and your immediate superior a source of stress to you?

Empowering Leadership:

1. Does your immediate superior encourage you to participate in important decisions?
2. Dies your immediate superior encourage you to speak up when you have different opinions?
3. Does your immediate superior help you develop your skills?

Supportive Leadership:

1. If needed, can you get support and help with your work from you immediate superior?
2. If needed, is you immediate superior willing to listen to your work-related problems?
3. Are you achievements appreciated by your immediate superior?

**Response scale:**

5-point Likert scale, 1= Vey seldom or never, 2= Rather seldom, 3= Sometimes, 4= Rather often, 5= Very often or always.

## Hopkins Symptoms Check List (HSCL-10) Items:

"Listed below are some symptoms or problems that people sometimes have. Please read each one carefully and decide how much the symptoms bothered or distressed you during the last week, including today:

1. Suddenly scared for no reason
2. Feeling fearful
3. Faintness, dizziness, or weakness
4. Feeling tense of keyed up
5. Blaming yourself for things
6. Difficulty in falling asleep or staying asleep
7. Feeling blue
8. Feeling of worthlessness
9. Feeling everything is an effort
10. Feeling hopeless about the future"

**Response scale:**
4-point Likert scale, 1= Not at all, 2= A little bit, 3= Quite a bit, 4= Extremely
